# Okbyungpoongsan (Yupingfeng) for treating allergic rhinitis: A protocol for the systematic review of controlled trials

**DOI:** 10.1097/MD.0000000000013227

**Published:** 2018-11-09

**Authors:** Minseong Lee, Youngjo Kim, Ju Ah Lee

**Affiliations:** College of Korean Medicine, Gachon University, Seongnam, Korea.

**Keywords:** allergic rhinitis, Okbyungpoongsan, protocol, systematic review, Yupingfeng

## Abstract

**Background:**

Okbyungpoongsan (OBPS) is widely used as a treatment for allergic rhinitis (AR) in Far East countries. Many clinical trials have assessed the efficacy and safety of the OBPS formula for treating AR. Here, we systematically will review the clinical evidence for and against administration of OBPS.

**Methods and analysis:**

All RCTs of decoctions or modified decoctions will be included. The methodological quality of the RCTs will be analyzed using the Cochrane Collaboration tool for assessing risk of bias, while confidence in the cumulative evidence will be evaluated using the Grading of Recommendations Assessment, Development and Evaluation (GRADE) instrument.

**Ethics and dissemination:**

This systematic review will be published in a peer-reviewed journal and will also be disseminated electronically and in print. The review will be updated to provide additional information and guide healthcare practices.

Registration number: CRD42017080292.

## 1 Introduction

Allergic rhinitis (AR) is a prevalent inflammatory disease that affects the upper respiratory tract. Non-infectious rhinitis is caused by IgE-mediated hypersensitivity to allergens.^[1]^ Most allergens are inhalation antigens, such as house dust mites (HDMs), farina, grass, trees, and pollen. Symptoms of rhinitis can be characterized as nasal obstructions, sneezing, and rhinorrhea. Additionally, itchy nose, sore throat, itchy eyes, watery eyes, cough, and headache are observed.^[2]^ These symptoms tend to be more intense in spring and autumn.^[3]^

Due to a high incidence of symptoms, AR induces work absenteeism. Thus, work productivity can be diminished and impaired.^[4]^ The prevalence of AR is estimated to be 15% to 25% and is surely increasing.^[5]^ In addition, AR is associated with variable respiratory diseases, such as asthma, sinusitis, conjunctivitis, and nasal polyposis. Therefore, the direct and indirect costs of AR to society are considerable.^[6]^

Mast cell stabilizers, histamine H1 receptor antagonists, leukotriene receptor antagonists, and Th2 cytokine inhibitors are generally used to abate allergic reactions of nasal mucous, and intranasal or oral steroids are effective at reducing severe AR symptoms, such as nasal obstruction.^[7]^ However, adverse effects of these therapeutic agents, such as hepatic and gastrointestinal disorders, sleepiness, jaundice, nasal irritation, rash, or diarrhea, are continuously reported.^[8]^ Additionally, surgical treatment can be another option for patients who are resistant to drugs or have nasal deformities,^[9]^ but the collagen fibre nets that occupy the lamina propria and nasal glands, eosinophils, and venous plexus, as well as the number of IgE+ cells, are reduced after surgery.^[10]^ In locations where broad-spectrum antibiotics are prescribed more often, specifically extended-spectrum cephalosporins and macrolides, the rates of multidrug-resistant pneumococcal disease are higher.^[5]^

Antibiotics are prescribed at more than 100 million adult ambulatory care visits annually, and 41% of these prescriptions are for respiratory conditions.^[1]^ Inappropriate antibiotic use for ARTI is an important contributor to antibiotic resistance, which is an urgent public health threat. As a result, it is necessary to develop a method that can prevent the adverse effects of therapeutic agents or surgical treatments for AR and reduce its symptoms. Okbyungpoongsan (OBPS) is widely used in traditional Korean medicine (TKM) and traditional Chinese medicine (TCM) for treating the symptoms of AR. In this study, we systematically will review randomized controlled trials (RCTs) to assess the effectiveness and safety of OBPS for the treatment of AR.

## 2 Methods

### 2.1 Study registration

This study will follow the guidelines outlined in the Preferred Reporting Items for Systematic Reviews and Meta-Analysis (PRISMA) statement for meta-analyses of healthcare interventions;^[11]^ additionally, the protocol adheres to the PRISMA Protocols (PRISMA-P).^[12]^ The protocol for this systematic review has been registered on PROSPERO 2017 under the number CRD42017080292.

### 2.2 Ethical approval

Because this study was not a clinical study, ethical approval was not required.

### 2.3 Data sources

The following databases will be searched from inception to the present date: MEDLINE, EMBASE, the Cochrane Central Register of Controlled Trials (CENTRAL), AMED, and CINAHL. We will also search Korean medical databases and 3 Chinese databases, including CNKI (the China Academic Journal, the China Doctoral Dissertations and Master's Theses Full-text Database, the China Proceedings of Conference Full-Text Database and the Century Journal Project), Wanfang and VIP. In addition, we will search a Japanese database and conduct non-electronic searches of conference proceedings.

## 3 Types of studies

Prospective RCTs that evaluate the effectiveness of OBPS (Yu Ping Feng San [YPFS]) for AR will be included in this review. Both treatment with OBPS (YPFS) alone and concurrent treatment with OBPS (YPFS) and another therapy will be considered to be acceptable if OBPS (YPFS) is applied to the intervention group only and any other treatment is equally provided to other groups. Trials with any type of control intervention will be included. No language restrictions will be imposed. Hard copies of all of the articles will be obtained and read in full.

## 4 Types of participants

All strains of AR will be eligible for inclusion. Participants who have both AR and accompanying diseases will be excluded. There will be no restrictions based on other conditions, such as age, sex, or symptom severity.

## 5 Types of interventions

Studies that evaluate any type of formulation (i.e., decoction, tablet, pill, or powder) of OBPS (YPFS) will be eligible for inclusion. The compositions of interventions will be reviewed, and interventions involving herbal combinations that differ from the original OBPS (YPFS) from the perspective of traditional East Asian medicine will be excluded from this review.

## 6 Data extraction and quality assessment

Hard copies of all of the articles will be obtained and read in full. Two authors (ML and YK) will perform the data extraction and quality assessment using a predefined data extraction form. Risk of bias will be assessed using the Cochrane Handbook risk of bias assessment tool version 5.1.0, which considers random sequence generation, allocation concealment, blinding of participants and personnel, blinding of outcome assessment, incomplete outcome data, selective reporting and other sources of bias.^[13]^ The results of the assessments will be presented using scores of “L”, “U”, and “H”, with “L” indicating a low risk of bias, “U” an uncertain risk of bias and “H” a high risk of bias. Disagreements will be resolved by discussion among all of the authors. When disagreements regarding selection cannot be resolved through discussion, an arbiter (JAL) will make the final decision.

## 7 Data collection and synthesis

### 7.1 Outcome measures

#### 7.1.1 Primary outcome

Improved effectiveness including the total treatment efficacy; that is, the number of patients whose AR symptoms improved was the primary outcome.

#### 7.1.2 Secondary outcomes

Adverse events.

Change in symptoms (e.g, Response rate).

Information related to OBPS (YPFS) usage.

Pattern type of the response based on TKM or TCM therapy.

Range of the dosage of OBPS (YPFS) in each study.

### 7.2 Assessment of bias in the included studies

We will independently assess the bias of the included studies according to the criteria in the Cochrane Handbook, version 5.1.0; these criteria included random sequence generation, allocation concealment, blinding of participants and personnel, blinding of outcome assessment, incomplete outcome data, selective reporting and other sources of bias.^[13]^

### 7.3 Data synthesis

Differences between the intervention and control groups will be assessed. Mean differences (MDs) with 95% confidence intervals (CIs) will be used to measure the effects of the treatments for continuous data. We will convert other forms of data into MDs. For outcome variables on different scales, we will use standard MDs (SMDs) with 95% CIs. For dichotomous data, we will present treatment effects to be relative risk ratios (RRs) with 95% CIs; other binary data will be converted into RR values.

All statistical analyses will be conducted using Cochrane Collaboration's software programme Review Manager (RevMan) version 5.3. (Copenhagen, the Nordic Cochrane Centre, the Cochrane Collaboration, 2014) for Windows. We will contact the corresponding authors of studies with missing information to acquire and verify the data whenever possible. When appropriate, we will pool data across studies to conduct a meta-analysis using fixed or random effects. We will use GRADEpro software from Cochrane Systematic Reviews to create a Summary of Findings table.

### 7.4 Unit of analysis issues

For crossover trials, data from the first treatment period will be used. For trials that assessed more than 1 control group, the primary analysis will combine data from each control group. Subgroup analyses of the control groups will be performed. Each patient will be counted only once in the analyses.

### 7.5 Addressing missing data

Intention-to-treat analyses, including all randomized patients, will be performed. For patients with missing outcome data, a last observation carry-forward analysis will be performed. When individual patient data are initially unavailable, we will review the original source or the published trial reports for these data.

### 7.6 Assessment of heterogeneity

Based on the data analysis, we will use random- or fixed-effects models to conduct the meta-analysis. Chi-squared and I^2^ tests will be used to evaluate the heterogeneity of the included studies. I^2^ values >50 will indicate high heterogeneity. When heterogeneity is observed, subgroup analyses will be conducted to explore the possible causes.^[14]^

### 7.7 Assessment of reporting biases

Funnel plots will be generated to detect reporting biases when a sufficient number of included studies (at least 10 trials) is available.^[15]^ However, as funnel plot asymmetries are not equivalent to publication biases, we will aim to determine the possible reasons for any asymmetries in the included studies, such as small-study effects, poor methodological quality, and true heterogeneity.^[15,16]^

## 8 Discussion

OBPS has long been used in East Asian countries and has been mentioned in numerous books. OBPS consists of 3 herbal ingredients: Atractylis Rhizoma (18.75 g), Ledebouriellae Radix (9 g), and Astragali Radix (9 g). OBPS has shown efficacy against allergic reactions and inflammation and thus has been applied to treat AR, COPD, asthma, and so on.^[2,17,18]^ OBPS regulates inflammation processes by both boosting inflammation and suppressing inflammation. In detail, OBPS stimulates mRNA and protein expression of pro-inflammatory cytokines via NF-kB and suppresses mRNA and protein expression in a lipopolysaccharide (LPS)-induced chronic inflammation model.^[3]^ Clinical trials involving OBPS considered various forms of OBPS, such as decoctions, granules, drop pills, and syrup. All types of OBPS will be included in this study. There was 1 research article that was a systematic review and meta-analysis of RCTs.^[2]^ This article included 22 RCTs, and OBPS was compared to placebo and pharmacotherapy. However, the study had some limitations, as mentioned in the article itself. This systematic review provides updated information on the effectiveness of OBPS and suggests a range of dosages, modifications, and durations that could improve its effectiveness. The original OBPS formula was composed of 3 herbs, which are mentioned above; however, primary studies showed a certain range of diversity in their compositions based on TCM or TKM theory. Therefore, we will investigate the composition of each formula used in the primary studies.

## Author contributions

ML and YK conceived the study, developed the criteria, searched the literature, analyzed the data, and wrote the protocol. JAL revised the manuscript. All authors have read and approved the final manuscript.

**Conceptualization:** Ju Ah Lee.

**Funding acquisition:** Ju Ah Lee.

**Writing – original draft:** Minseong Lee, Youngjo Kim.

**Writing – review & editing:** Ju Ah Lee.

## References

[1] Small M Piercy J Demoly P Burden of illness and quality of life in patients being treated for seasonal allergic rhinitis: a cohort survey. Clin Transl Allergy 2013;3:33. 24107462 10.1186/2045-7022-3-33 PMC3852977

[2] Luo Q Zhang CS Yang L Potential effectiveness of Chinese herbal medicine Yu ping feng san for adult allergic rhinitis: a systematic review and meta-analysis of randomized controlled trials. BMC Complement Altern Med 2017;17:485. 29110710 10.1186/s12906-017-1988-5 PMC5674829

[3] Du CY Choi RC Zheng KY Yu Ping Feng San, an ancient Chinese herbal decoction containing Astragali Radix, Atractylodis Macrocephalae Rhizoma and Saposhnikoviae Radix, regulates the release of cytokines in murine macrophages. PLoS One 2013;8:e78622. 24244327 10.1371/journal.pone.0078622 PMC3823765

[4] Yoo KH Ahn HR Park JK Burden of respiratory disease in korea: an observational study on allergic rhinitis, asthma, copd, and rhinosinusitis. Allergy Asthma Immunol Res 2016;8:527–34. 27582404 10.4168/aair.2016.8.6.527 PMC5011053

[5] Passali D Cingi C Staffa P The international study of the allergic rhinitis survey: outcomes from 4 geographical regions. Asia Pac Allergy 2018;8:e7. 29423374 10.5415/apallergy.2018.8.e7 PMC5796967

[6] Blaiss MS Allergic rhinitis: direct and indirect costs. Allergy Asthma Proc 2010;31:375–80. 20929603 10.2500/aap.2010.31.3329

[7] Seidman MD Gurgel RK Lin SY Clinical practice guideline: allergic rhinitis. Otolaryngol Head Neck Surg 2015;152 suppl 1:S1–43. 10.1177/0194599814561600 25644617

[8] Okubo K Kurono Y Ichimura K Japanese guidelines for allergic rhinitis 2017. Allergol Int 2017;66:205–19. 28214137 10.1016/j.alit.2016.11.001

[9] Brozek JL Bousquet J Agache I Allergic rhinitis and its impact on asthma (ARIA) guidelines-2016 revision. J Allergy Clin Immunol 2017;140:950–8. 28602936 10.1016/j.jaci.2017.03.050

[10] Mori S Fujieda S Igarashi M Submucous turbinectomy decreases not only nasal stiffness but also sneezing and rhinorrhea in patients with perennial allergic rhinitis. Clin Exp Allergy 1999;29:1542–8. 10520084 10.1046/j.1365-2222.1999.00645.x

[11] Liberati A Altman DG Tetzlaff J The PRISMA statement for reporting systematic reviews and meta-analyses of studies that evaluate health care interventions: explanation and elaboration. PLoS Med 2009;6:e1000100. 19621070 10.1371/journal.pmed.1000100 PMC2707010

[12] Shamseer L Moher D Clarke M Preferred reporting items for systematic review and meta-analysis protocols (PRISMA-P) 2015: elaboration and explanation. BMJ 2015;349:10.1136/bmj.g7647 25555855

[13] Higgins JPT Altman DG Sterne JAC Chapter 8: Assessing risk of bias included studies. 2011 In: Cochrane Handbook for Systematic Reviews of Interventions Version 5.1.0 [updated March 2011]. The Cochrane Collaboration, 2011. Available from http://handbook.cochrane.org.

[14] Deeks JJ Higgins JPT Altman DG Chapter 9: Analysing data and undertaking meta-analyses. In: Cochrane Handbook for Systematic Reviews of Interventions Version 5.1.0 [updated March 2011]. The Cochrane Collaboration, 2011 Available from http://handbook.cochrane.org.

[15] Sterne JAC Egger M Moher D Chapter 10: Addressing reporting biases. In: Cochrane Handbook for Systematic Reviews of Interventions Version 5.1.0 [updated March 2011]. The Cochrane Collaboration, 2011 Available from http://handbook.cochrane.org.

[16] Egger M Davey Smith G Schneider M Bias in meta-analysis detected by a simple, graphical test. BMJ 1997;315:629–34. 9310563 10.1136/bmj.315.7109.629 PMC2127453

[17] Yang ZS Yan JY Han NP Anti-inflammatory effect of Yu-Ping-Feng-San via TGF-beta1 signaling suppression in rat model of COPD. Iran J Basic Med Sci 2016;19:993–1002. 27803787 PMC5080430

[18] Liu X Shen J Fan D Yupingfeng San Inhibits NLRP3 Inflammasome to Attenuate the Inflammatory Response in Asthma Mice. Front Pharmacol 2017;8:00944. 10.3389/fphar.2017.00944 PMC5743824 29311942

